# Three-dimensional image study of accelerated maxillary expansion in oral breathing kids

**DOI:** 10.1016/j.bjorl.2022.01.007

**Published:** 2022-02-15

**Authors:** Fauze Ramez Badreddine, Lucia Hatsue Yamamoto, Andre Besen, Daniela Pimentel Machado Renófio Hoppe, Reginaldo Raimundo Fujita, Mario Cappellette Junior

**Affiliations:** a Universidade Federal de São Paulo (UNIFESP), Departamento de Otorrinolaringologia e Cirurgia de Cabeça e Pescoço, Disciplina de Otorrinolaringologia Pediátrica, São Paulo, SP, Brazil; b Universidade Federal de São Paulo (UNIFESP), Departamento de Otorrinolaringologia e Cirurgia de Cabeça e Pescoço, Disciplina de Otorrinolaringologia Pediátrica, Especialista em Ortodontia e Ortopedia Facial, São Paulo, SP, Brazil

**Keywords:** Maxillary hypoplasia, Rapid maxillary expansion, Nose, Soft tissue changes, Mouth breathing

## Abstract

**Objective:**

To evaluate, by a three-dimensional study, the volumetric and integumentary effects of rapid maxillary expansion on the nose, in mouth breathing kids with maxillary hypoplasia, in the short term, assessing the possible interference of gender, growth and age on the results achieved.

**Methods:**

120 mouth breathing patients with maxilla hypoplasia were divided into an Experimental Group treated by rapid maxillary expansion (n = 104, 62 males and 42 females, mean age 10.1 years, SD = 2.10, ranging from 5.1 to 13.9 years); and Control Group, constituted by 16 patients (9 males and 7 females, mean age 9.3 years, SD = 2.1 years, ranging from 6.1 to 13.2 years). Patients in the experimental group underwent multislice computed tomography examinations at two different times: (T1) pre-expansion and (T2) post-expansion. The control group was submitted to the same tests at the same time intervals. Six soft tissue variables of the nose were studied, besides the volume and area of the nasal cavity, and the measurement and comparison of data between T1 and T2 were performed using the Dolphin Imaging 11.7 Premium software.

**Results:**

The experimental group showed significant mean increases in all soft tissue variables studied (*p* < 0.005), yet there were no significant changes in the control group. In the comparison between groups, only inclination of the nasal dorsum did not present any significant change.

**Conclusion:**

Rapid maxillary expansion may alter the nasal shape and physiology, by anatomical changes in the nose soft tissues, making it an important aid in the treatment of mouth breathing in childhood.

**Level of evidence:**

The soft tissues of the nose play an important role in nasal shape and physiology and facial esthetics, and since they are directly related to the nasal valves, they are fundamental for maintenance and stability of the nasal breathing pattern.

## Highlights

• Rapid maxillary expansion can change the shape and physiology of the nose.
• The soft tissues of the nose are fundamental for maintenance and stability of the nasal breathing pattern.
• Soft tissues are directly related to the nasal valves.

## Introduction

Mouth breathing indicates the presence of respiratory obstacles that can cause several functional changes, such as imbalance of the oral and perioral muscles, maxillary narrowing or hypoplasia, lip deficiency, tongue lowering^1^, ^2^, ^3^ and other anomalies that interfere with the correct growth and development of the face and occlusion and the entire stomatognathic system,^4^, ^5^, ^6^ besides favoring the appearance of several respiratory diseases in the pediatric population, such as chronic rhinitis and Obstructive Sleep Apnea (OSAS).^6^, ^7^, ^8^, ^9^

The evaluation of nasal obstruction is a routine aspect in otolaryngological practice^10^ and, over the years, orthodontics and facial orthopedics have proven to be an important aid in the treatment of children with impairment of correct transverse development of the maxilla during facial growth. The maxilla constitutes almost 50% of the anatomical structure of the Nasal Cavity (NC)^11^; thus, it is believed that, due to the relationship between the nasal floor and the maxillary base,^12^, ^13^ the transverse adequacy of this structure may favor the patient with improvement in respiratory medical conditions, by changes in nasal shape and physiology.^6^, ^8^, ^12^, ^13^, ^14^, ^15^

Rapid Maxillary Expansion (RME), standardized by Haas in the 1960s,^16^ is still considered the orthodontic/orthopedic procedure of choice for the treatment of maxillary hypoplasia and, since then, orthodontists and otolaryngologists have evaluated the possible effects of this procedure on the NC.^4^, ^7^, ^12^, ^13^ However, even though the literature strongly reports that RME may increase the nasal permeability, reducing the upper airway resistance,^4^, ^6^, ^7^, ^12^^,^^13^, ^17^, ^18^ several investigators, for different reasons, state that post-RME respiratory improvements are subjective and that the actual changes induced in the upper airway, specifically in the nose, are still controversial and inconclusive.^7^, ^8^, ^9^, ^12^, ^19^

Much of the still present skepticism occurs due to lack of evidence on the behavior of nose soft tissues after RME. Skeletal changes have been and are still extensively explored; however, it is believed that possible soft tissue changes, especially in the anterior region of the nose,^14^, ^17^, ^18^, ^20^, ^21^, ^22^ due to the close relationship with the nasal valves,^12^, ^23^, ^24^ may provide more important and evident data on the efficiency of RME in aiding the treatment of mouth breathing than information obtained only by skeletal changes.

## Methods

The study was conducted on 120 mouth breathing patients with maxillary hypoplasia and indication for RME. The sample was divided in Experimental Group (EG) consisting of 104 patients (62 males and 42 females, mean age 10.1 years, SD = 2.10, range 5.1 to 13.9 years); and Control Group (CG) formed by 16 patients (9 males and 7 females, mean age 9.3 years, SD = 2.1 years, range 6.1–13.2 years), assessed from a pre-existing database. Characterization of the sample regarding gender and age presented in Table 1 shows that there are no significant differences in age between EG and CG (*p* = 0.128).

**Table 1:** Characterization of patients according to gender and age.

Variables	Total (n = 120)	EG (n = 104)	CG (n = 16)	*p*
Gender				
Female – n (%)	49 (40.8%)	42 (40.4%)	7 (43.8%)	0.799^a^
Male – n (%)	71 (59.2%)	62 (59.6%)	9 (56.3%)	
Age (years)				
Minimum	5.1	5.1	6.1	0.128^b^
Maximum	13.9	13.9	13.2	
Mean	10.0	10.1	9.3	
Standard deviation	2.1	2.1	2.2	

a Significance value of the Chi-Square test.

b Significance value of the Student’s *t*-test for independent samples.

Patients with allergic rhinitis (clinically treated or untreated), syndromes or craniofacial abnormalities, as well as patients with obstructed upper airway undergoing or not to adenotonsillectomy surgery were excluded from the study. The study was approved by the Institutional Review Board under nº 0079/2017 and registered in Clinical Trials (ID: CRB-ORTO3).

Patients in the EG were treated with the Hyrax expander and submitted to the same activation protocol, with 6/4 initial activation and 2/4 daily activations until achievement of compatibility of the upper alveolar bone base with the lower WALA edge (area of greater transverse width of the mandibular arch in its buccal face). The expanders were kept in place for a period of 6 months and were removed after radiographic confirmation of new bone formation in the mid palatal suture.

Patients in the EG underwent CT exams at two different times: (T1) pre-RME and (T2) after expander removal. The participants in CG were also submitted to CT exams in the same period as patients in the EG. All EG and CG CT scans were performed in the same place (environment with temperature control (21 °C) and relative humidity), using the same equipment (multislice Philips® Brillance CT scanner 64 channels), respecting the ALARA principle.

This study comprised a global evaluation of the sample and an evaluation segmented by gender and age to verify possible influences of gender dimorphism and growth in the results. The nasal structures were manipulated and measured using the Dolphin*®* Imaging image manipulation software (Version 11.7 Premium) facilitating the segmentation of the NC and achievement of its volume, allowing assessment in the three dimensions, besides reconstruction of sections that allowed measurement of the analyzed structures.

The CT images do not follow an equality pattern regarding the head positioning between CT scans of times T1 and T2, thus, to ensure more accurate measurements, the heads were repositioned before measurements, using specific software tools, following recommended methodologies.^25^, ^26^

**Figure 1:**
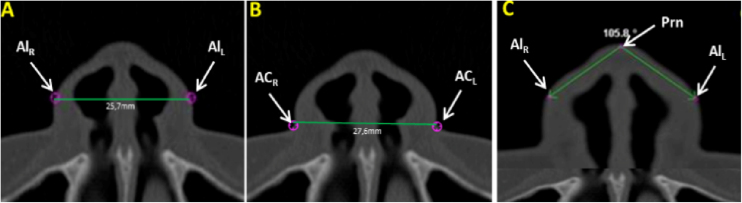
Axial multiplanar images illustrating: (A) Alar Width (Al_R_–Al_L_), (B) Soft tissue Insertion Width (AC_R_–AC_L_), (C) Alar Inclination Angle (AC_R_-Prn. AC_L_-Prn).

**Figure 2:**
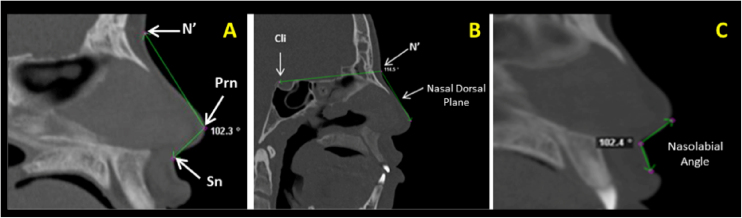
Sagittal multiplanar images illustrating: (A) Nose tip angle (N’-Prn. Prn-Sn), (B) Nasal dorsum angle (Cli-N’. N’-Dorsum plane), (C) Nasolabial angle (formed by the lower edge of the nose and upper lip margin).

Measurements of the nose soft tissue were obtained in axial (Fig. 1A‒C) and sagittal (Fig. 2A‒C) images of multiplanar sections, evaluating the following structures: Alar Width (AW) by the linear distance (mm) between points Al_r_ and Al_l_ (Fig. 1A), Soft tissue insertion width (STIW) by the linear distance (mm) between points Ca_r_ and Ca_l_ (Fig. 1B), Angle of Alar Inclination (Alar.Inc), by the angle formed between line Alr-Prn and line Al_l_-Prn (Fig. 1C), Nose tip angle (Nose tip), by the angle formed between line N’-Prn and line Prn-Sn (Fig. 2A), Nasal Dorsum angle (N.D), by the angle formed between line Cli-N’ and line N’-Dorsum plane (Fig. 2B) and Nasolabial Angle (NAW) by the angle formed between the inferior nose border (base) and lip philtrum (upper lip margin) (Fig. 2C).

**Figure 3:**
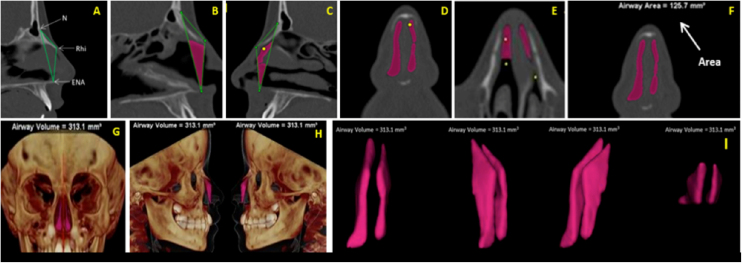
(A) Sagittal multiplanar image illustrating the anterior skeletal segmentation of NC through the anatomical landmarks of choice. (B‒C) Filling of NC to obtain volume and area, on the sagittal multiplanar image on the right and left sides, respectively. (D‒E) Filling of NC to obtain volume and area in coronal and axial multiplanar images, respectively. (F) Achievement of the total area (ArT). (G‒H) Total volume obtained and illustrated in 3D reconstruction images in right and left coronal and sagittal views, respectively. (I) Image illustrating the total anterior volume of the NC, delimited by bone tissues, extracted from surrounding tissues.

Volume (VNT) and Area (ArT) measurements of the anterior nose region were measured in sagittal, axial, and coronal multiplanar images illustrated in Fig. 3A‒I. To assure the accuracy of volume measurements, the air radiation attenuation coefficient was adjusted before measurements using a phantom that was submitted to the same CT procedure as the research participants.

### Statistical analysis

To verify the adequacy of the sample, the comparison between pre-RME and post-RME in the EG, the sample (n = 104) assures the detection of small effects (d = 0.25), with a test power of 80% and significance level of 5% for the Student’s *t*-test for paired samples. In the CG the sample (n = 16) assures the identification of medium/large effects (d = 0.65) with a test power of 80%. The use of ANOVA to evaluate differences regarding age groups of the sample guarantees the detection of large effects (f = 0.39), with a test power of 80% and significance level of 5%.

The normality of data was studied by the Kolmogorov–Smirnov test. The results lead to non-rejection of the null hypothesis of the test, with a significance level of 5%, leading to the assumption that data have normal distribution. Based on that, the Student’s *t*-test for paired samples was used to compare pre-RME and post-RME measurements.

The comparison of independent groups (EG vs. CG, male vs. female) for continuous variables was performed using the Student’s *t*-test for independent samples. Analysis of Variance (ANOVA) was used to compare 3 or more independent groups (age group).

To compare EG with CG concerning gender, the chi-square independence test was used.

To study the method error, the Student’s *t*-test for paired samples showed no statistically significant differences (*p* > 0.05) between means of the first measurement and second measurement (repetition after 45 days), neither in repetitions by the same examiner nor in repetitions by the second examiner. These results assure excellent reliability of the measurements achieved.

## Results

Results of the global analysis of post-RME effects in EG and CG, as well as the analysis of effects between the two groups, are shown in Table 2.

**Table 2:** Comparison between pre-RME and post-RME values and between experimental (EG) and control (CG) groups of soft tissue measurements, volume, and area.

Variable	Group	Pre-RME (M ± SD)	Post-RME (M ± SD)	Change pre-RMEpost-RME (mean)		*p*^a^ (pre-RME – post-RME)
				Mean	%	
AW	EG	30.25 ± 3.24	32.37 ± 3.24	2.12	7.0%	**<0.001**
	CG	30.45 ± 1.90	30.51 ± 1.89	0.06	0.2%	0.590
p^b^ (between groups)		0.814	<0.001			
STIW	EG	31.86 ± 3.05	33.95 ± 2.85	2.09	6.5%	**<0.001**
	CG	31.86 ± 1.81	31.89 ± 1.89	0.03	0.1%	0.707
p^b^ (between groups)		0.992	0.002			
Alar.Inc	EG	101.50 ± 9.87	105.73 ± 9.57	4.23	4.2%	**<0.001**
	CG	104.61 ± 7.67	103.99 ± 7.63	−0.61	−0.6%	0.163
p^b^ (between groups)		0.231	0.004			
Nose tip	EG	108.90 ± 6.61	111.58 ± 6.43	2.68	2.5%	**<0.001**
	CG	112.23 ± 6.27	112.31 ± 6.21	0.08	0.1%	0.549
p^b^ (between groups)		0.061	0.004			
N.D	EG	112.59 ± 5.13	114.76 ± 4.90	2.17	1.9%	**<0.001**
	CG	111.65 ± 5.61	111.34 ± 5.49	−0.31	−0.3%	0.083
p^b^ (between groups)		0.500	0.012			
NLA	EG	107.62 ± 12.38	113.35 ± 12.26	5.73	5.3%	**<0.001**
	CG	117.34 ± 11.74	117.19 ± 11.82	−0.15	−0.1%	0.365
p^b^ (between groups)		0.004	0.003			

VNT	EG	636.66 ± 300.45	867.76 ± 310.47	231.10	36.3%	**<0.001**
	CG	498.63 ± 191.09	518.44 ± 175.38	19.81	4.0%	0.337
p^b^ (between groups)		0.078	<0.001			
ArT	EG	157.13 ± 50.62	200.28 ± 53.25	43.15	27.5%	**<0.001**
	CG	139.00 ± 30.49	140.38 ± 30.95	1.38	1.0%	0.151
p^b^ (between groups)		0.167	<0.001			

a *p* – Significance value of Student’s *t*-test for paired samples (differences between pre and post).

b *p* – Significance value of Student’s *t*-test for independent samples (differences between EG and CG).

The results of the global analysis showed that, in the EG, there were significant increases (*p* < 0.05) in all variables evaluated between moments T1 and T2, in which the Alar Width (AW) and the Soft Tissue Insertion Width (STIW) presented relevant increases of 7.0% and 6.5% respectively. Changes in Volume (VNT) and Area (ArT) also showed considerable increases of 36.3% and 27.5%, respectively. Among patients in the CG, no significant changes were identified between T1 and T2 (*p* > 0.05) and in the comparison between EG and CG, the most significant post-RME differences were found for AW, VNT and ArT (*p* < 0.001). Changes in nasal dorsum inclination (N.D) were not significant between groups (*p* = 0.012).

To verify the possible effects of gender and age on the results, controlling the possible effect of age differences and the different number of male and female patients, a random and balanced sample was selected in the EG regarding the distribution by gender and age (Table 3).

**Table 3:** Subsample balanced by gender and age group, in the EG.

Age group	Gender		Total per age range
	Female	Male	
5 to 7 years	n = 9	n = 9	n = 18
8 to 9 years	n = 10	n = 10	n = 20
10 to 11 years	n = 10	n = 10	n = 20
12 to 13 years	n = 10	n = 10	n = 20
Total per gender	n = 39	n = 39	n = 78

The results of comparison of changes by gender (Table 4) show that gender dimorphism did not have statistically significant influence on changes in any measurement analyzed.

**Table 4:** Comparison by gender concerning differences between pre- and post-RME, in the sample balanced by gender and age group, in the EG.

Variables (difference pre- and post-RME)	Female (n = 39)	Male (n = 39)	*p*^a^
	M ± SD	M ± SD	
Soft tissue measurements			
AW	2.13 ± 1.36	2.48 ± 2.20	0.399
STIW	2.25 ± 1.63	2.37 ± 2.24	0.796
Alar.Inc	4.11 ± 3.35	4.10 ± 3.52	0.992
Nose tip	2.55 ± 2.22	2.69 ± 2.90	0.810
N.D	2.18 ± 2.18	1.89 ± 1.56	0.501
NLA	5.54 ± 5.57	6.41 ± 4.78	0.462
Volume and area measurements			
VNT	239.91 ± 140.05	244.08 ± 115.58	0.887
ArT	48.37 ± 38.29	42.62 ± 26.75	0.444

a *p* – Significance value of Student’s *t*-test for independent samples.

The comparison of pre- and post-RME changes by age was studied using the sample balanced by gender and age group presented in Table 3. The results shown in Table 5 evidence that the greatest changes occurred in the age group 8–9 years (*p* ≤ 0.005) and the lowest results occurred between 12 and 13 years of age. These results allow to conclude that the greatest changes occur in younger patients, and that in the short term the growth factor has no influence on the results obtained for this sample.

**Table 5:** Comparison by age group regarding differences between pre-RME and post-RME, in the sample balanced by gender and age group, in the EG.

Variables (difference post-RME – pre-RME)	5‒7 years (n = 18)	8‒9 years (n = 20)	10‒11years (n = 20)	12‒13 years (n = 20)	*p*^a^
	M ± SD	M ± SD	M ± SD	M ± SD	
Soft tissue profile					
AW	2.09 ± 1.32	3.10 ± 2.73	2.04 ± 1.23	1.96 ± 1.44	**0.003**
STIW	2.37 ± 1.17	2.84 ± 3.26	2.00 ± 1.10	1.99 ± 1.37	**0.003**
Alar.Inc	4.54 ± 3.84	4.31 ± 3.49	3.49 ± 2.87	4.04 ± 3.50	**0.005**
Nose tip	2.47 ± 2.28	3.89 ± 3.56	2.45 ± 2.29	1.66 ± 1.23	**0.005**
N.D	2.54 ± 2.28	2.08 ± 1.81	1.71 ± 1.90	1.81 ± 1.47	0.516
NLA	6.89 ± 4.75	7.72 ± 5.05	4.73 ± 5.68	4.39 ± 4.73	**0.003**
Volume and area measurements					
VNT	226.05 ± 108.29	293.70 ± 163.76	225.75 ± 93.55	220.31 ± 128.88	**0.001**
ArT	46.44 ± 33.87	47.93 ± 27.68	45.20 ± 39.73	42.50 ± 31.78	**0.001**

a *p* – Significance value of ANOVA.

## Discussion

Since the first clinical reports on the role of RME in the growth and development of the face and occlusion, several investigators attempted to evaluate the effects of this procedure on the NC and the actual benefits to reestablish the normal nasal breathing pattern.^4^, ^7^, ^10^, ^11^, ^12^, ^13^, ^16^

Several studies and research have been and continue to be published stating that RME may alter the nasal shape and physiology,^6^, ^8^, ^12^, ^13^, ^14^, ^15^ allowing better respiratory response for the patient due to the proven enlargement in the NC base as a consequence of separation of the mid palatal suture. However, for many investigators, these data are still doubtful and inconclusive.^7^, ^8^, ^9^, ^12^, ^19^

Much of the skepticism still present is related to the fact that nearly all studies published to date have focused on skeletal alterations of the NC neglecting the importance of assessing the behavior of surrounding soft tissues that play an extremely important role in the maintenance and stability of results obtained post-RME,^14^, ^17^, ^18^, ^20^, ^21^, ^22^ besides their esthetic impact on the face.^21^, ^27^, ^28^

The soft tissues of the nose are directly related to the behavior of the nasal valves, which are considered the areas of greatest resistance to the airflow of the nose through the NC. In children, its collapse can be one of the main causes for the occurrence of mouth breathing.^12^, ^23^, ^24^, ^28^ Thus, the lack of scientific evidence about these structures raises many doubts about the effectiveness of RME to improve the respiratory pattern when only skeletal changes are evaluated.

The nose not only performs several vital functions, but also its shape, size and proportions provide important esthetic characteristics because it is located in the center of the face; thus, the width of nasal soft tissues, besides directly influencing the esthetic impact, can characterize areas of resistance in the NV region.^28^ However, according to the medical literature, not only the width but also the behavior of the nose tip in relation to its length and angulation also have great importance in nasal physiology,^24^, ^27^, ^28^ especially in patients with long nose and downs lanting tip, which may contribute to collapse of the valves, with consequent increase in the resistance of inspired airflow.^29^

Thus, this study aimed to investigate the soft tissue structures directly related to the base width of the soft tissue nose (alar width, soft tissue insertion width and alar inclination angle), projection and angulation of the nose tip (nose tip angle, nasal dorsum angle and nasolabial angle).

The few studies that address the nasal soft tissues use several evaluation methods such as photographs, direct measurements on the face or analysis of lateral or posteroanterior two-dimensional radiographs focusing mainly on changes in width. The present study comprised three-dimensional evaluations with the aid of CT; however, among the few studies that used similar evaluation methods, only the study of alar width and soft tissue insertion width are common in the methodologies. The present results revealed significant increases (*p* < 0.001) post-RME in the EG of 2.12 mm (7%) and 2.09 mm (6.5%) in Alar Width (AW) and Soft Tissue Insertion Width (STIW), respectively. Similar results for AW and STIW were found by Kim et al.^15^ (1.82 mm and 1.39 mm) and Yilmaz & Kucukkeles^22^ (1.69 mm and 1.16 mm). Pangrazio-Kulbersh et al.,^14^ evaluating only the AW, found non-significant increases of 1.78 mm. These studies conducted evaluations by CBCTs and small samples, as in the case of Kim et al.^15^ which had a study group of only eight patients. None of these studies included comparisons with a CG. In comparison with previous studies by our group using CT,^17^, ^18^ in the first study we observed post-RME changes of 1.13 mm and 1.43 mm for AW and STIW respectively, and in the second study increases of 1.36 mm and 1.62 mm. The results of both investigations were statistically significant and the difference in results with the present study may have been influenced by the number of individuals included in the EG (39 in study 1,^17^ 30 in study 2,^18^ 104 in the present study), besides the software used to measure the evaluated structures. Greater results, also with the aid of CT, were obtained for AW in a study conducted by Magnusson et al.^21^ (2.88 mm), yet in adult patients, by surgically assisted RME.

The results obtained in this study for the alar inclination angle with an increase of 4.23° (4.2%) combined to the changes obtained in AW and STIW measurements, show how RME alters the width of nose soft tissues and can help in reducing the resistance of nasal airflow.

The nasolabial angles, nose tip and nasal dorsum are related to rotation and projection of the nose tip, and upward rotation (angle increase) can cause a higher tip with shorter dorsum, as well as a downward rotation (decreased angle) may indicate longer dorsum and down slanting tip in which the decrease in muscle tone may contribute to collapse of the nasal valves.^27^, ^28^, ^29^, ^30^, ^31^

The results obtained in this research showed significant increases of 2.68° (2.5%), 2.17° (1.9%) and 5.73° (5.3%) for the nose tip, nasal dorsum, and nasolabial angles, respectively, showing the tendency to anterior and ascending projection of the nose tip after RME. Even though elevation of the nose tip is considered more favorable, its excess (upturned nose) can also lead to poor airflow direction and can cause respiratory disorders.^28^ Thus, we also aimed to evaluate the changes in area and volume after RME. The significant mean increases of 36.3% in total nasal volume and 27.5% in total area showed that the soft tissue changes obtained were beneficial for the reduction of nasal respiratory resistance in this sample.

Other points of doubt and discussion regarding the effects of RME in the NC are the fact that all studies in the literature address the results, in the short term, in a global sample, i.e., regardless of gender and age. For some authors, gender can influence the results due to the greater skeletal framework of male patients; however, male patients with younger age have smaller skeletal structure than older females. Thus, in a global sample, several authors find it difficult to state that gender influences the results obtained after RME, since the stages of growth and development of the NC can be different between the study participants.

To try to reduce the bias and look for more accurate results regarding growth and gender dimorphism, the sample was segmented to make a comparative analysis between male and female patients within the same age group. Since the global sample of EG (n = 104) had more male patients (n = 62), we segmented the sample (n = 78) to obtain the same number of patients of both genders 39 males and 39 females) within the same age range, as described in Table 5.

The results showed that there are no significant differences between genders in the different age groups (*p* > 0.005) (Table 4). However, the comparison of results obtained between age groups (Table 5) indicates that the younger the patient, the larger the changes, showing that for this sample, in the short term, there is no influence of growth and that the earlier the treatment, the greater the chances of decreasing the nasal air resistance.

The greatest limitation of this study may be the impossibility of accompanying patients until adulthood to verify the stability of results in the long term. However, despite the lack of this information, it can be concluded that the early treatment of maxillary hypoplasia using RME brings encouraging perspectives regarding the aid of this procedure in improving or even solving mouth breathing, preventing several respiratory diseases in childhood that could worsen in adulthood. Also, this type of study can assist in proper planning of esthetic procedures in the nose tip and base and also collaborate for the achievement of objective measurements in early or late surgical results.^17^

It is important to emphasize that patients in the CG were not harmed in relation to their general and oral health condition. After the T2 period, nine of the sixteen patients were properly treated following the same protocols as patients in EG. The remaining seven patients formally gave up treatment.

## Conclusion

1 Rapid maxillary expansion significantly increased all soft tissue measurements, as well as the volume and area of the NC, suggesting that, in the short term, the procedure may be an important aid in the treatment of mouth breathing in young individuals with maxillary hypoplasia.
2 Gender does not interfere with the results obtained in the short term
3 The earlier the treatment, the better the prognosis regarding the real benefits of RME on nasal shape and physiology.

## Conflicts of interest

The authors declare no conflicts of interest.
